# Supplementary material for: Non-canonical Wnt signalling modulates the endothelial shear stress flow sensor in vascular remodelling
Source: eLife. 2016 Feb 4;5:e07727. doi: 10.7554/eLife.07727 (PMC4798962; doi:10.7554/eLife.07727)
Supplement: Supplementary file 1. — DOI: http://dx.doi.org/10.7554/eLife.07727.017 [file elife-07727-supp1.doc]

**Supplementary File 1: List of antibodies used in the immunochemistry and immunofluorescence studies**

| **Antibody** | **Manufacturer** | **Cat. No.** | **Dilution** |
| --- | --- | --- | --- |
| Cleaved Caspase 3 | Cell Signaling | 9661 | 1:400 |
| Collagen IV | AbD Serotec | 2150-1470 | 1:400 |
| Erg1/2/3 | Santa Cruz Antibodies | sc-353 | 1:200 |
| GM130 | BD Pharmingen | 610822 | 1:400 |
| Golph4 | Abcam | Ab28049 | 1:400 |
| ICAM2 | BD Pharmingen | 553326 | 1:200 |
| Lef1 | Cell Signaling | 2230 | 1:100 |
| NG2 | Millipore | AB5320 | 1:400 |
| Donkey a-Rabbit Alexa488 | Invitrogen | A-21206 | 1:400 |
| Donkey a-Rabbit Alexa594 | Invitrogen | A-21207 | 1:400 |
| Donkey a-Rabbit Alexa647 | Invitrogen | A-31573 | 1:400 |
| Goat a-Mouse Alexa568 | Invitrogen | A-21124 | 1:400 |
| Goat a-Rat Alexa555 | Invitrogen | A-21434 | 1:400 |
| Donkey a-Rat Alexa647 | Invitrogen | A-21247 | 1:400 |
| Donkey a-Rabbit Fab fragment | Jackson’s Laboratories | 711-007-003 | 1:100 |
| IsolectinB4 – Alexa647 | Invitrogen | I-32450 | 1:500 |
