# Supplementary material for: Non-canonical Wnt signalling modulates the endothelial shear stress flow sensor in vascular remodelling
Source: eLife. 2016 Feb 4;5:e07727. doi: 10.7554/eLife.07727 (PMC4798962; doi:10.7554/eLife.07727)
Supplement: Supplementary file 2. — DOI: http://dx.doi.org/10.7554/eLife.07727.018 [file elife-07727-supp2.doc]

**Supplementary File 2: List PCR primers used in Wnt ligand gene expression profiling of isolated retinal endothelial cells**

| **Gene** | **Primer F** | **Primer R** |
| --- | --- | --- |
| Wnt1 | 5’- CTCTTCGGCAAGATCGTCA | 5’- TAGTCGCAGGTGCAGGACTC |
| Wnt2 | 5’- CCAACGAAAAATGACCTCGT | 5’- GGGAAGTCAAGTTGCACACA |
| Wnt2b | 5’- CTGCTGCTGCTACTCCTGACT | 5’- GGGGATGTTGTCACAGATCA |
| Wnt3 | 5’- CTTCTAATGGAGCCCCACCT | 5’- GAGGCCAGAGATGTGTACTGC |
| Wnt3a | 5’- GTGGCTGAGGGTGTCAAAG | 5’- GGTGGCTTTGTCCAGAACAG |
| Wnt4 | 5’- CCTGCGACTCCTCGTCTTC | 5’- TCTGGATCAGGCCTTTGAGT |
| Wnt5a | 5’- ATTGTCCCCCAAGGCTTAAC | 5’- CTCCCGGGCTTAATATTCCA |
| Wnt5b | 5’- AGCAGGTGACAGAACCGTCTTTCT | 5’- TGGAAATCCACAACCAGTGGGAGA |
| Wnt6 | 5’- CGGTAGAGCTCTCAGGATGC | 5’- GTAGGATCCATGACCAAGGG |
| Wnt7a | 5’- TACACAATAACGAGGCGGGT | 5’- CCTTGAGCACGTAGCCTAGC |
| Wnt7b | 5’- ACGTGTTTCTCTGCTTTGGC | 5’- CCAGGCCAGGAATCTTGTT |
| Wnt8a | 5’- GGTGGAATTGTCCTGAGCAT | 5’- GGTGACTGCGTACATGATGG |
| Wnt8b | 5’- CTGCCTTTCTCCGAAGACC | 5’- ACCAGGTAAGCCTTTGGACC |
| Wnt9a | 5’- GATGCTGGATGGGTCCCT | 5’- GGGAGGATAGTCAGGGGTTC |
| Wnt9b | 5’- CGAGGAGATGCGAGAGTGC | 5’- GGAAGGGTGTCAGGACCTC |
| Wnt10a | 5’- CATGAGTGCCAGCATCAGTT | 5’- GCACTCTCTCGAAAACCTCG |
| Wnt10b | 5’- TTCACGAGTGTCAGCACCA | 5’- AGGAGAAAGCACTCTCACGG |
| Wnt11 | 5’- TCCAGCCTATTGGCAGACATCACA | 5’- AGCAGGACCGATTCCCTCTTTCAA |
| Wnt16 | 5’- AGCAGAGTCCTGCTAGCCAT | 5’- TTTTCCAGCAGGTTTTCACA |
