# Supplementary material for: Non-canonical Wnt signalling modulates the endothelial shear stress flow sensor in vascular remodelling
Source: eLife. 2016 Feb 4;5:e07727. doi: 10.7554/eLife.07727 (PMC4798962; doi:10.7554/eLife.07727)
Supplement: Supplementary file 3. — DOI: http://dx.doi.org/10.7554/eLife.07727.019 [file elife-07727-supp3.doc]

**Supplementary File 3: List qPCR Taqman primers used in gene expression experiments.**

| **Primer** | **Cat. No.** |
| --- | --- |
| mouse Wls/Gpr177/Evi | Mm00509695 |
| mouse Dscr1 | Mm01213406 |
| mouse Vegfa | Mm00437304 |
| mouse Kdr/Vegfr2 | Mm00440099 |
| mouse Cdh5 | Mm00486938 |
| mouse Pecam1 | Mm01242584 |
| mouse Notch1 | Mm00435245 |
| mouse Dll4 | Mm00444619 |
| mouse Hey2 | Mm00469280 |
| mouse Nrarp | Mm00482529 |
| mouse Ctnnb1/beta-Catenin | Mm00483033 |
| mouse Lef1 | Mm00550265 |
| mouse Ccnd1/CyclinD1 | Mm01334599 |
| mouse Axin2 | Mm00443610 |
| mouse Nrp1 | Mm00435379 |
| mouse Flt1 | Mm00438980 |
| mouse Tie2 | Mm00443243 |
| mouse Tie1 | Mm00495643 |
| mouse Robo4 | Mm00452963 |
| mouse Unc5b | Mm00504054 |
| mouse EphrinB2 | Mm01215897 |
| mouse EphB4 | Mm01201157 |
| mouse Gapdh | Mm00516104 |
| mouse Klf2 | Mm00500486 |
| mouse Klf4 | 4352339E |
| mouse Ptgs2 | Mm00478374 |
| human Wnt5a | Hs00998537 |
| human Wnt11 | Hs00182986 |
| human Vegfr2 | Hs00182986 |
| human Pecam1 | Hs00182986 |
| human Cdh5 | Hs00182986 |
| human Ptgs2/Cox2 | Hs00182986 |
| human Klf2 | Hs00182986 |
| human Klf4 | Hs00182986 |
| human Gapdh | 4326317E |
